# A robust screening method for dietary agents that activate tumour-suppressor microRNAs

**DOI:** 10.1038/srep14697

**Published:** 2015-10-01

**Authors:** Keitaro Hagiwara, Luc Gailhouste, Ken Yasukawa, Nobuyoshi Kosaka, Takahiro Ochiya

**Affiliations:** 1 Division of Molecular and Cellular Medicine, National Cancer Center Research Institute, 5-1-1, Tsukiji, Chuo-ku, Tokyo 104-0045, Japan; 2 Department of Biological Sciences, Tokyo Institute of Technology, 4259 Nagatsuta-cho, Midori-ku, Yokohama 226-8501, Japan; 3 Integrative Bioscience and Biomedical Engineering, Graduate School of Science and Engineering, Waseda University, 2-2 Wakamatsu, Shinjuku, Tokyo 162-8480, Japan

## Abstract

Certain dietary agents, such as natural products, have been reported to show anti-cancer effects. However, the underlying mechanisms of these substances in human cancer remain unclear. We recently found that resveratrol exerts an anti-cancer effect by upregulating tumour-suppressor microRNAs (miRNAs). In the current study, we aimed to identify new dietary products that have the ability to activate tumour-suppressor miRNAs and that therefore may serve as novel tools for the prevention and treatment of human cancers. We describe the generation and use of an original screening system based on a luciferase-based reporter vector for monitoring miR-200c tumour-suppressor activity. By screening a library containing 139 natural substances, three natural compounds — enoxolone, magnolol and palmatine chloride — were identified as being capable of inducing miR-200c expression in breast cancer cells at 10 μM. Moreover, these molecules suppressed the invasiveness of breast cancer cells *in vitro*. Next, we identified a molecular pathway by which the increased expression of miR-200c induced by natural substances led to ZEB1 inhibition and E-cadherin induction. These results indicate that our method is a valuable tool for a fast identification of natural molecules that exhibit tumour-suppressor activity in human cancer through miRNA activation.

Diet is one of the most important environmental factors that can modulate gene expression and affect animal physiology^1^. The use of natural products for the treatment of various diseases such as diabetes, obesity and cancer has also been reported^2^,^3^,^4^. Indeed, more than 50% of the drugs currently available are natural substances or related compounds^5^. *In vitro* screening using human cancer cell lines enabled the classification of these products according to their cytotoxicity^6^. For instance, active molecules such as paclitaxel were characterised and appeared to be useful for clinical applications. However, although anti-cancer agents show cytotoxic effect against most cancer cells, resistant populations of cells may survive, leading to recurrence or metastasis. Therefore, finding new and more effective anti-cancer agents is still a real necessity for the development of innovative therapeutic protocols.

MicroRNAs (miRNAs) are small, non-coding RNAs that repress the expression of a wide variety of genes at the post-transcriptional level via sequence-specific base pairing to the 3′ UTR region of multiple target mRNAs^7^. Recent studies have reported that miRNAs can fine-tune in various biological processes, including development, organogenesis, metabolism and homeostasis^8^. It has also been reported that miRNA deregulation can be associated with cancer development, progression and metastasis^9^,^10^.

Numerous studies have demonstrated that the knockdown of oncogenic miRNAs or the reexpression of tumour-suppressor miRNAs can significantly promote drug sensitivity, inhibit cancer cell proliferation and suppress invasion and metastasis^11^,^12^,^13^. Kong and collaborators reported that natural products are involved in signal transduction^14^. However, it remains unclear how these molecules might regulate miRNA expression. We previously provided evidence that resveratrol and pterostilbene suppress cancer cell malignancy *in vitro* and *in vivo* through the transcriptional activation of tumour-suppressor miRNAs^15^. These findings provide evidence that dietary intake of natural agents can have beneficial effects on human physiology and survival by modulating miRNA biogenesis. However, it is still necessary to identify potent natural substances that activate tumour-suppressor miRNAs. Here, we have described a relevant experimental approach for the screening of natural products with the ability to induce tumour-suppressor miRNAs. By using this method, three compounds— enoxolone, magnolol and palmatine chloride — were highlighted and shown to be capable of upregulating the expression of the tumour-suppressor miRNA miR-200c in MCF7 cells. Moreover, we demonstrated that these three molecules promote miR-200c-dependent anti-cancer effects in breast cancer cells. These results demonstrate that our novel screening method allows the identification of small molecules, such as chemical compounds, peptides, proteins and oligonucleotides, that can activate tumour-suppressor miRNAs in breast cancer.

## Results

### Establishment of a screening system for monitoring miRNA activity

To identify natural compounds that activate tumour-suppressor miRNAs, we designed a reporter system to monitor miR-200c activity. We selected miR-200c to serve as a reporter in this study because we previously demonstrated that resveratrol suppresses breast cancer cell malignancy by increasing the expression of miR-200c, and we provided evidence of the tumour-suppressor activity of this specific miRNA^15^.

**Figure 1:**
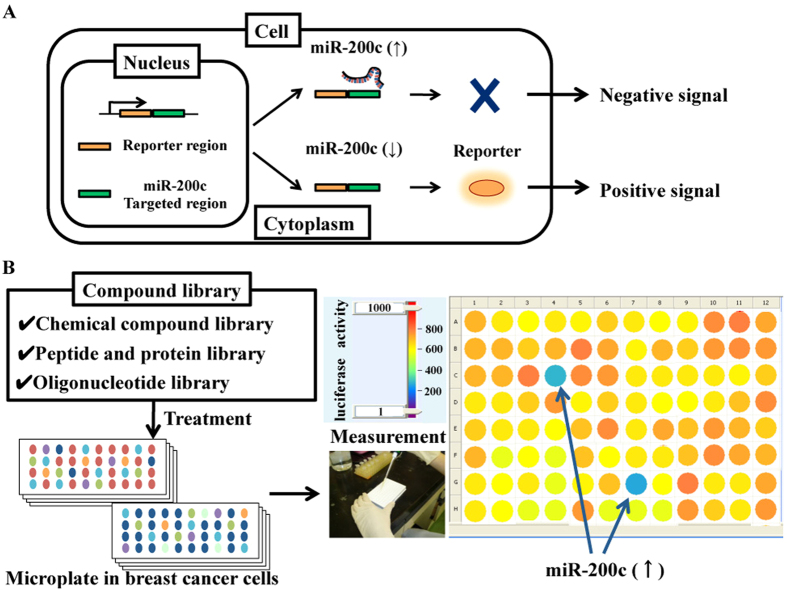
Screening method for the identification of molecules that promote miR-200c activity. (**A**) Schematic representation of the miR-200c monitoring system. To monitor miR-200c activity, we generated a reporter sensor vector that expresses a version of firefly luciferase and contains tandem-binding sites with a precise complementary sequence to miR-200 c. If the level of miR-200c increases after treatment with a natural substance, the firefly luciferase activity decreases. (**B**) A graphical scheme of the screening method for the characterisation of small molecules that can activate miR-200c expression. Using this experimental system, the compounds contained in our natural product library were screened. MCF7 cells stably expressing the reporter sensor vector were treated with 139 natural substances at concentrations of 10 μM. After 2 days of exposure, the cells were harvested and the firefly luciferase activity was measured.

As shown in Fig. 1A, our sensor vector expresses a version of firefly luciferase that contains a sequence complementary to miR-200c in its 3′ UTR region. This vector also encodes *Renilla* luciferase as a control reporter for normalisation. In this system, firefly luciferase activity decreases when miR-200c activity is increased and vice versa (Fig. 1B).

### Natural product screening to identify agents that enhance miR-200c activity

**Figure 2:**
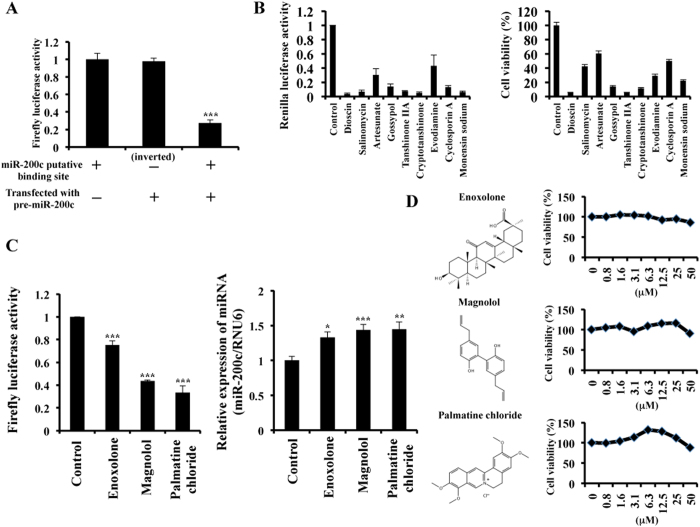
Identification of natural products that activate miR-200c expression. (**A**) MCF7 cells stably expressing firefly luciferase and *Renilla* luciferase (pmiR-200c-MCF7) were transfected with 100 nM pre-miR-200c or AllStars Negative Control for 2 days. Whole-cell lysates were collected and firefly luciferase activity was measured and normalised to *Renilla* luciferase activity using the Dual-Glo Luciferase Assay System. The values on the *y-axis* are depicted relative to the firefly luciferase activity of the AllStars Negative Control transfectant, which is defined as 1.0. (**B**) pmiR-200c-MCF7 cells were seeded and treated with natural compounds (10 μM) or DMSO (Control) for 2 days. Whole-cell lysates were collected, and *Renilla* luciferase activity was measured (left panel). The values on the *y-axis* are depicted relative to the *Renilla* luciferase activity of the DMSO treatment (Control), which is defined as 1.0. After 3 days of culture, cell viability was measured by the MTS assay (right panel). The values on the *y-axis* are depicted relative to the cell viability of the DMSO (Control) treatment, which is defined as 100. (**C**) pmiR-200c-MCF7 cells were cultured and treated with enoxolone, magnolol and palmatine chloride at 10 μM for 2 days. Whole-cell lysates were collected, and firefly luciferase activity was measured and normalised to *Renilla* luciferase activity using the Dual-Glo Luciferase Assay System (left panel). The values on the *y-axis* are depicted relative to the firefly luciferase activity of the DMSO treatment (Control), which is defined as 1.0. Cell extracts were also subjected to qRT-PCR (right panel). The values on the *y-axis* are depicted relative to the miR-200c expression in the DMSO-treated cells (Control), which is defined as 1.0. (**D**) The chemical structures of enoxolone, magnolol and palmatine chloride are shown in the left panels. pmiR-200c-MCF7 cells were cultured and treated with enoxolone, magnolol and palmatine chloride (10 μM) at 10 μM for 3 days. Cell viability was examined using the MTS assay (right panels). The values on the *y-axis* are depicted relative to the cell viability of the DMSO (Control) treatment, which is defined as 100. All data are shown as the mean ± S.E. *P < 0.05, **P < 0.01, ***P < 0.001.

Next, we generated two stable cell lines: one expresses the sensor vector pmiR-200c-MCF7, and the other expresses the control vector pmiR-control-MCF7. As shown in Fig. 2A, transfection with miR-200c mimic significantly downregulated the firefly luciferase activity in pmiR-200c-MCF7 cells compared with transfection with the control miRNA. By contrast, firefly luciferase activity was not altered in response to miR-200c induction in pmiR-control-MCF7 cells (Fig. 2A).

Using this experimental system, a collection of 139 natural substances was screened (Selleck Chemicals, Houston, TX) (Supplementary Table S1). We identified 9 molecules — dioscin, salinomycin, artesunate, gossypol, tanshinone IIA, cryptotanshinone, evodiamine, cyclosporin A and monensin sodium salt — that significantly inhibited the *Renilla* luciferase activity in pmiR-200c-MCF7 cells 48 h after treatment (Fig. 2B). Moreover, we found that these 9 compounds dramatically inhibited cell growth at 10 μM (Fig. 2B).

Then, from our screening data, we selected three substances — enoxolone, magnolol and palmatine chloride — that strongly induced miR-200c expression (Supplementary Table S1, Fig. 2C). Importantly, these three molecules did not show any cell toxicity on MCF7 until a concentration of 50 μM (Fig. 2D). These data suggest that our screening method is suitable for identifying natural substances with miR-200c activation capacity in breast cancer cells. To examine the possible effects of these three agents on the expression of other tumour-suppressor miRNAs, we performed qRT-PCR. As a result, several tumour-suppressor miRNAs appeared upregulated following treatment with natural products. For instance, we found that palmatine chloride treatment increased the tumour-suppressor miRNAs miR-34a and miR-141 in MCF7 cells (Supplementary Fig. S1).

### The natural compounds enoxolone, magnolol and palmatine chloride show a miR-200c-dependent anti-cancer activity

**Figure 3:**
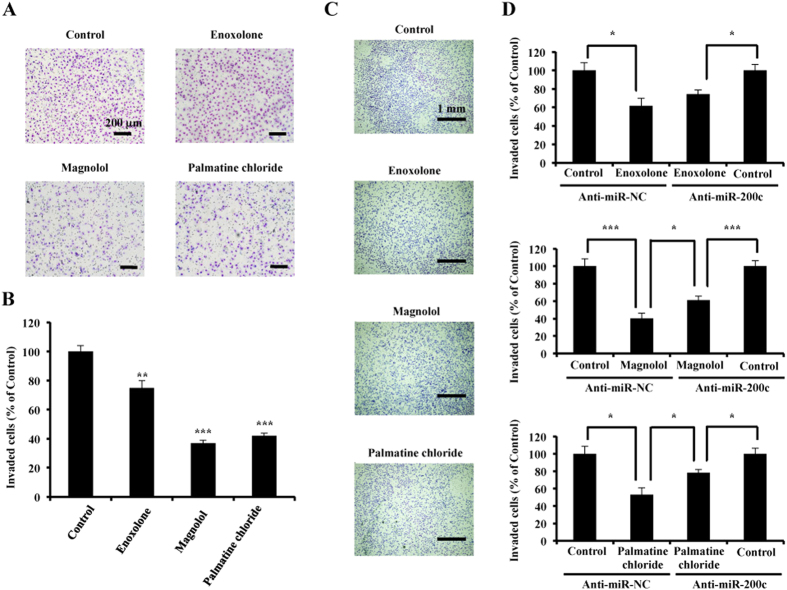
Inhibition of invasive activity through the enhancement of miR-200c activity by natural substances. (**A,B**) MDA-MB-231-luc-D3H2LN cells were grown and treated with enoxolone, magnolol or palmatine chloride (10 μM), or DMSO (Control), for 1 day and subjected to a Matrigel invasion assay. Representative photographs (**A**) and quantification (**B**) are shown. Scale bar: 200 μm. (**C,D**) MDA-MB231-luc-D3H2LN cells were grown and transiently transfected with anti-miR-200c or anti-miR-NC (Control). After 4 hours, the cells were treated with enoxolone, magnolol or palmatine chloride (10 μM), or DMSO (Control), for 1 day and subjected to a Matrigel invasion assay. Representative photographs (C) and quantification (D) are shown. Scale bar: 1 mm. All data are shown as the mean ± S.E. *P < 0.05, **P < 0.01, ***P < 0.001.

A recent study demonstrated that miR-200c strongly inhibits the invasion ability of breast cancer cells^16^. To examine whether the three selected compounds can influence invasiveness, we performed invasion assays using the highly invasive breast cancer cell line MDA-MB-231-luc-D3H2LN. We found that enoxolone, magnolol, and palmatine chloride reduced the invasion capacity of these cells (25%, 63% and 58% inhibition, respectively) compared with the control (DMSO) (Fig. 3A,B).

To confirm that the invasiveness inhibition induced by these three substances is regulated by miR-200c, MDA-MB-231-luc-D3H2LN cells were transfected with an antisense oligonucleotide targeting miR-200c (miR-200c inhibitor) in the presence of these molecules. As shown in Fig. 3C,D, the tumour-suppressor effect of each natural compound was abrogated by the addition of miR-200c inhibitor as the invasiveness of the MDA-MB-231-luc-D3H2LN cells increased. Taken together, these results demonstrate that the anti-cancer effect of these natural products is mediated by the induction of miR-200c.

### Inhibition of ZEB1 and enhancement of E-cadherin expression through miR-200c induction by enoxolone, magnolol or palmatine chloride

ZEB1 (zinc-finger E-box-binding homeobox 1) is an important EMT activator in human breast cancer. It has been reported that the overexpression of ZEB1 promoted the metastasis of colorectal cancer in a mouse xenograft model^17^. In addition, miR-200c activation inhibits ZEB1 expression, resulting in E-cadherin induction in breast cancer cells^18^. We hypothesised that the induction of miR-200c by natural products may have a similar effect on ZEB1 and E-cadherin expression, leading to tumour suppression.

**Figure 4:**
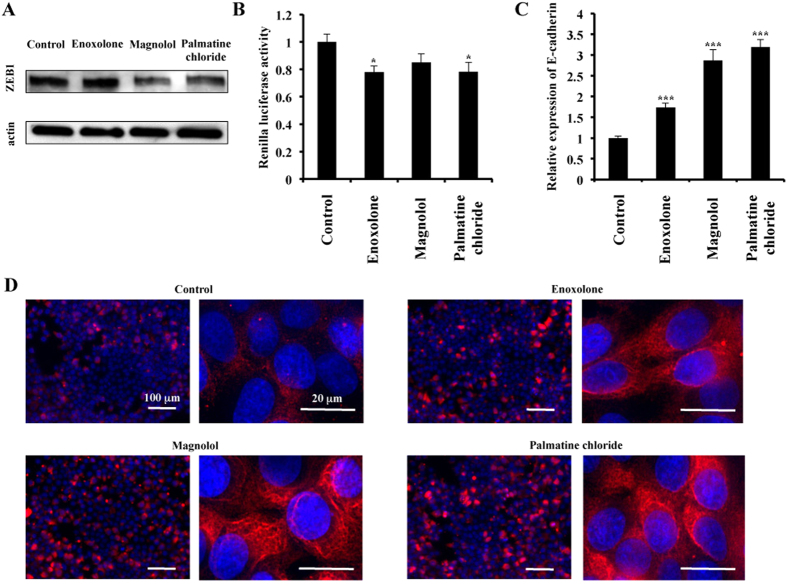
Effect of natural compounds on breast cancer cell phenotypes. (**A**) MDA-MB-231 cells were grown and treated with enoxolone, magnolol or palmatine chloride (10 μM), or DMSO (Control). After 2 days of culture, the cells were collected, and proteins were extracted with M-PER. ZEB-1 expression was detected using immunoblotting. β-actin was used as a loading control. (**B**) MDA-MB-231 cells were grown and transiently transfected with a ZEB-1 3′ UTR or psiCheck-2 vector (Control) prior to treatment with the three natural compounds. After 2 days of culture, the cells were subjected to a *Renilla* luciferase reporter assay. The values on the *y-axis* are depicted relative to the *Renilla* luciferase activity of the cells treated with DMSO (Control), which is defined as 1.0. (**C**) MCF7 cells were grown and treated with enoxolone, magnolol or palmatine chloride (10 μM), or DMSO (Control). After 2 days of culture, cell extracts were subjected to qRT-PCR. The values on the *y-axis* are depicted relative to the E-cadherin expression of the cells treated with DMSO (Control), which is defined as 1.0. (**D**) Immunofluorescence staining of E-cadherin (red) was performed following 2 days of treatment. Nuclei are shown in blue. Lower magnification views are shown in the left panels, and the bars indicate 100 μm. Higher magnification views are shown in the right panels, and the bars indicate 20 μm. All data are shown as the mean ± S.E. *P < 0.05, ***P < 0.001.

First, our results showed that magnolol and palmatine chloride treatment suppressed ZEB1 expression in MDA-MB-231 cells, whereas enoxolone had no effect (Fig. 4A). Then, to validate that miR-200c directly modulates ZEB1 protein levels, we performed ZEB1 3′ UTR assays. We found that enoxolone, magnolol and palmatine chloride downregulated *Renilla* luciferase activity after cells were transfected with a plasmid containing the ZEB1 3′ UTR (Fig. 4B). This result indicates that, in response to treatment with natural substances, the increased expression of miR-200c leads to ZEB1 inhibition through direct targeting of its 3′ UTR.

To examine the effect of natural products on E-cadherin expression, we performed qRT-PCR and immunofluorescence staining of E-cadherin in MCF7 cells after treatment with enoxolone, magnolol or palmatine chloride. As shown in Fig. 4C, all compounds induced E-cadherin expression in MCF7 cells. Furthermore, immunofluorescence staining showed that natural products significantly increased E-cadherin-positive cells compared with the control (DMSO) (Fig. 4D).

**Figure 5:**
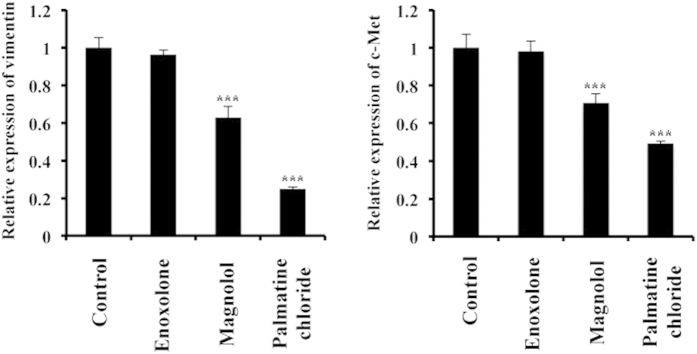
Effect of natural compounds on EMT markers. MCF7 cells were grown and treated with enoxolone, magnolol or palmatine chloride (10 μM), or DMSO (Control). After 2 days of culture, cell extracts were subjected to qRT-PCR. The values on the *y-axis* are depicted relative to the mRNA expression of vimentin (left panel) and c-Met (right panel). Cells treated with DMSO (Control) were used as controls, and their values were defined as 1.0. All data are shown as the mean ± S.E. *P < 0.05, ***P < 0.001.

Lastly, we analysed the expression of several EMT markers following natural product treatment^19^. Globally, the EMT markers that we assessed appeared downregulated by one or more natural products after treatment. The most relevant results were obtained with vimentin and c-Met, as their expression was significantly decreased following magnolol and palmatine chloride treatment (Fig. 5). Taken together, these results suggest that these natural compounds could contribute to the prevention of breast cancer via the modulation of the miR-200c/ZEB1/E-cadherin pathway.

## Discussion

New cancer therapies may be developed via the targeting of aberrantly expressed miRNAs, and the administration of specific tumour-suppressor miRNAs has shown beneficial effects for cancer patients^20^. Recent studies have reported that specific natural products, including resveratrol, EGCG, curcumin and isoflavone, can modulate the expression profile of miRNAs^15^,^21^,^22^,^23^. These findings suggest that miRNA regulation using natural products represents a promising strategy for human cancer treatment. In this study, we identified three agents — enoxolone, magnolol and palmatine chloride —that upregulate miR-200c expression. Enoxolone, also known as 18β-glycyrrhetinic acid, is a pentacyclic triterpenoid from liquorice root that exerts anti-tumour activity through the induction of apoptosis in breast cancer^24^. In addition, enoxolone can repress cell invasiveness and metastasis by inhibiting PI3K/Akt-mediated NF-kB activity^25^. Magnolol has been isolated from the bark of Magnoliaceae family members, and is used as a herbal medicine to treat several diseases or symptoms such as coughs, acute pain, and gastrointestinal disorders in East Asia^26^. Moreover, magnolol inhibits breast cancer invasiveness by suppressing MMP-9 expression^27^. Finally, palmatine chloride has a structure that is similar to that of berberine alkaloids. This natural product is present in Coptidis Rhizoma and has been used as a traditional medicine in East Asia^28^. Palmatine chloride is known for exerting anti-malarial effects^29^. Hambright and collaborators reported that palmatine inhibits cancer cell proliferation and invasion through decreased activation of NFkB^30^.

Although the natural substances that we identified have previously been reported as anti-cancer agents, their effects on tumour-suppressor miRNA activation are still mostly unknown. Consequently, the development of comprehensive methods for the screening of small molecules appears to be extremely valuable for the characterisation of new beneficial substances and determining the mechanisms by which these substances control tumour-suppressor miRNA expression. For instance, our results showed that palmatine chloride treatment inhibited invasiveness and controlled the expression of miR-34a and one of its targets, the c-Met oncogene. The oncogenic activity of this receptor tyrosine kinase has been extensively reported in various types of cancer, where it has an important role in cancer growth, invasion and metastasis^31^,^32^. Interestingly, other natural products such as (–)-oleocanthal, which is derived from olive oil, can inhibit HGF-induced c-Met activation and its invasive properties in breast cancer^33^. In addition, Kim and collaborators reported that miR-34a directly represses the expression of c-Met and can regulate the downstream ERK2/MAPK signaling pathway^34^. These data show that natural products can specifically repress cell invasion by indirectly inhibiting the expression of a group of tumour suppressors that still need to be further characterised.

In this study, we decided to use miR-200c for screening because the tumour-suppressor function of this miRNA has been extensively reported by several teams, including our group^15^,^16^,^18^. However, we cannot exclude the possibility that natural products may also influence the expression of other tumour-suppressor miRNAs. For example, our previous study revealed that resveratrol treatment was associated with increased levels of multiple tumour-suppressor miRNAs^15^. To address this point, we globally analysed the expression of miRNAs in response to palmatine chloride treatment and found that this compound increased a number of tumour-suppressor miRNAs, including miR-34a and miR-141 (Supplementary Fig. S1). These 2 tumour-suppressor miRNAs have been previously well characterised. Specifically, miR-34a directly down-regulates the expression of BCL-2 and SIRT1, inhibiting the proliferation and migration of breast cancer cells^35^. In addition, miR-141 strongly suppresses cancer cell migration and invasion by reducing TGFβ2^16^. These observations confirm the consistency of our screening method for the detecting natural substances that efficiently activate tumour-suppressor miRNAs.

We developed an original method that shows potential for high-throughput screening of natural products with tumour-suppressor miRNA up-regulation property. Indeed, in the case of conventional methods such as qRT-PCR, at least 6 hours are needed to measure the expression of miRNAs whereas our method is faster and easier, and can be completed within 1 hour using a small number of cells. Moreover, our method can be performed in 96-well plates, which is a requirement for high-throughput screening of large-compound libraries.

In conclusion, our study reports a valuable screening strategy for the identification of small molecules that can activate tumour-suppressor miRNAs. Using the tumour-suppressor miRNA miR-200c as a reporter, we demonstrated that enoxolone, magnolol and palmatine chloride can have positive effects on cells by downregulating oncogenes. This process occurs via an miRNA-based regulatory mechanism. Indeed, miR-200c upregulation after treatment with one of these compounds inhibited ZEB1 expression, resulting in E-cadherin induction and vimentin inhibition in breast cancer cell lines. Taken together, these results suggest that enoxolone, magnolol and palmatine chloride have an important role in breast cancer prevention by upregulating miR-200c. Although further investigations are needed to characterise the mechanism of action of these natural products in human cancers and clarify how these compounds can modulate the expression of tumour-suppressor miRNAs, our strategy could be valuable for identifying new drugs with curative potential by modulating tumour-suppressor miRNA expression. Importantly, this method is not limited to tumour-suppressor miRNAs and could be applied to the characterisation of miRNA-related natural products that have roles in development, differentiation and disease.

## Methods

### Reagents

The antibiotic solution (containing 10,000 U/mL penicillin and 10 mg/mL streptomycin), the trypsin-EDTA mixture (containing 0.05% trypsin and EDTA), FBS (foetal bovine serum) and donkey anti-goat Alexa 594 were obtained from Invitrogen (Carlsbad, CA, USA). Goat polyclonal anti-E-cadherin (s-17, sc-31020) was obtained from Santa Cruz Biotechnology (Santa Cruz, CA, USA). Rabbit monoclonal anti-ZEB1 (D80D3) was purchased from Cell Signaling Technology (Danvers, MA, USA). Hoechst 33258 was obtained from Dojindo (Kumamoto, Japan). Enoxolone (G10105), magnolol (M3445) and palmatine chloride (361615) were purchased from Sigma-Aldrich (St Louis, MO, USA).

### Natural Product Library

The Natural Product Library was purchased from Selleck Chemicals (Houston, TX, USA). This library contains a collection of 139 natural compounds supplied as solutions dissolved in DMSO.

### Plasmids

The pmiRGLO Dual-Luciferase miRNA Target Expression Vector was purchased from Promega (Madison, WI, USA). For the miR-200c reporter assay, pmiRGLO-200c was constructed by introducing tandem-binding sites with a sequence that is perfectly complementary to miR-200c into the multiple cloning site of the pmirGLO vector at the XhoI and SalI sites. The sequences of the binding sites are as follows: 5′- AAACCTAGACTCGAGCCACATTACCCGGCAGTATTAAAGAATTCTTTCCATCATTACCCGGCAGTATTAGTCGACTGGCCGCAA -3′ (sense) and 5′- TTGCGGCCAGTCGACTAATACTGCCGGGTAATGATGGAAAGAATTCTTTAATACTGCCGGGTAATGATGGACTCGAGTCTAGGTTT -3′ (antisense).

### Cell culture

MDA-MB-231 cells (American Type Culture Collection), MCF7 cells (American Type Culture Collection) and MDA-MB-231-luc-D3H2LN cells (Xenogen, Alameda, CA) were cultured in RPMI 1640 (Invitrogen, Carlsbad, CA, USA) containing 10% heat-inactivated FBS and the antibiotic solution at 37 °C in 5% CO_2_.

### Establishment of stable cell lines

Stable MCF7 cell lines used to monitor miR-200c activity were generated by selection with geneticin (500 μg/mL). MCF7 cells were transfected with 0.5 μg of the pmirGLO vector at 90% of confluency in 24-well dishes using Lipofectamine LTX reagent in accordance with the manufacturer’s instructions. Twelve hours after transfection, the cells were replated in a 15-cm dish, followed by a 3-week selection with the antibiotic. Ten surviving single colonies were selected from each transfection and were then cultured for 2 additional weeks.

### Cell proliferation assay (MTS assay)

A total of 5,000 cells per well were seeded in 96-well plates. The following day, the cells were treated with natural products. Three days after culturing, cell viability was measured using the Cell Counting Kit-8 (Dojindo, Kumamoto, Japan) in accordance with the manufacturer’s instructions. Absorbance was measured at 450 nm using the EnVision system (Wallac, Turku, Finland).

### Screening of a library containing 139 natural substances

MCF7 cells stably expressing our firefly luciferase and *Renilla* luciferase reporter vector (pmiR-200c-MCF7) were seeded onto 96-well plates at a density of 10,000 cells per well. The following day, the cells were treated with 139 natural substances at 10 μM. After 2 days, the cells were harvested and the firefly luciferase activity was measured and normalised to the *Renilla* luciferase activity. All assays were performed in triplicate and repeated at least three times. Representative data are shown in the manuscript.

### Transwell invasion assay

Breast cancer cell invasion was assayed in 24-well BioCoat Matrigel Invasion Chambers (8 μm; BD Biosciences Pharmingen, San Diego, CA, USA) in accordance with the manufacturer’s protocol. Briefly, cells were treated with natural products. The following day, 20,000 cells were plated in the upper chamber, which contained RPMI 1640 without FBS. The lower chambers were filled with RPMI 1640 containing 10% FBS as a chemoattractant. Twenty-two hours later, the non-invasive (upper chamber) cells were removed with a cotton swab. Cells that migrated through the membrane to the lower surface of the membrane were fixed with methanol and stained with Diff Quick staining (Sysmex, Kobe, Japan). For quantification, the cells were observed microscopically and counted in four random fields. All assays were performed in triplicate. Invasive values were normalised to the values obtained from cells treated with DMSO.

### Isolation of miRNAs and quantitative real-time PCR (qRT-PCR)

Total RNAs were extracted from cultured cells using the QIAzol and miRNeasy Mini Kit (Qiagen, Valencia, CA, USA) in accordance with the manufacturer’s protocol. PCR was performed in 96-well plates using the 7300 Real-Time PCR System (Applied Biosystems, Foster City, CA, USA). All reactions were performed in triplicate. TaqMan qRT-PCR kits and human E-cadherin and human β-actin TaqMan Expression Assays were purchased from Applied Biosystems (Foster City, CA, USA). Reverse transcription (Applied Biosystems, Foster City, CA, USA) and TaqMan quantitative PCR (Applied Biosystems, Foster City, CA, USA) were performed in accordance with the manufacturer’s instructions. SYBR Green I qRT-PCR was performed, and the β-actin housekeeping gene used to normalise the variation in the cDNA levels. The following pairs of primers were used for gene amplification: for vimentin, 5′-TCTGGATTCACTCCCTCTGG-3′ (forward) and 5′-GGTCATCGTGATGCTGAGAA-3′ (reverse); for c-Met, 5′-CAGGCAGTGCAGCATGTAGT-3′ (forward) and 5′-GATGATTCCCTCGGTCAGAA-3′ (reverse); and for β-actin, 5′-ACTCTTCCAGCCTTCCTTCC-3′ (forward) and 5′-AGCACTGTGTTGGCGTACAG-3′ (reverse).

### Immunofluorescent staining

After washing three times with PBS (–), the cells were fixed in cold methanol (Wako, Japan). Then, the cells were incubated in RPMI 1640 containing E-cadherin primary antibodies at a dilution of 1:100 for 60 min and subsequently incubated in RPMI 1640 containing Alexa Fluor fluorescent secondary antibodies. Nuclei were visualised using Hoechst 33258 (Dojindo, Kumamoto, Japan) staining for observation under a confocal microscope (FluoView FV1000; Olympus, Tokyo, Japan).

### Statistical analysis

The data are presented as the mean ± S.E.M. Each experimental point is the average of at least triplicates. All experiments were repeated at least 3 times. Statistical analyses were performed using Student’s *t*-test.

## Additional Information

**How to cite this article**: Hagiwara, K. *et al.* A robust screening method for dietary agents that activate tumour-suppressor microRNAs. *Sci. Rep.* **5**, 14697; doi: 10.1038/srep14697 (2015).

## Supplementary Material

Supplementary Information

## References

[b1] Lee C.-K., Klopp R. G., Weindruch R. & Prolla T. A. Gene expression profile of aging and its retardation by caloric restriction. Science. 285, 1390–1393 (1999). 10464095 10.1126/science.285.5432.1390

[b2] Coleman D. L., Kuzava J. E. & Leiter E. H. Effect of diet on incidence of diabetes in nonobese diabetic mice. Diabetes. 39, 432–436 (1990). 2318346 10.2337/diab.39.4.432

[b3] Davis C. D. & Ross S. A. Dietary components impact histone modifications and cancer risk. Nutr Rev. 65, 88–94 (2007). 17345961 10.1111/j.1753-4887.2007.tb00285.x

[b4] Epstein L. H., Myers M. D., Raynor H. A. & Saelens B. E. Treatment of pediatric obesity. Pediatrics. 101, 554–570 (1998). 12224662

[b5] Newman D. J. & Cragg G. M. Natural Products as Sources of New Drugs over the Last 25 Years. J. Nat Prod. 70, 461–477 (2007). 17309302 10.1021/np068054v

[b6] Perdue R. E. Jr. Procurement of plant materials for antitumor screening. Cancer Treat Rep. 60, 987–998 (1976). 991157

[b7] Hammell C. M., Lubin I., Boag P. R., Blackwell T. K. & Ambros V. nhl-2 Modulates microRNA activity in Caenorhabditis elegans. Cell. 136, 926–938 (2009). 19269369 10.1016/j.cell.2009.01.053 PMC2670343

[b8] Kwak P. B., Iwasaki S. & Tomari Y. The microRNA pathway and cancer. Cancer Sci. 101, 2309–2315 (2010). 20726859 10.1111/j.1349-7006.2010.01683.x PMC11159795

[b9] Calin G. A. *et al.* Frequent deletions and down-regulation of micro- RNA genes miR15 and miR16 at 13q14 in chronic lymphocytic leukemia. Proc Natl Acad. Sci. 99, 15524–15529 (2002). 12434020 10.1073/pnas.242606799 PMC137750

[b10] Calin G. A. *et al.* Human microRNA genes are frequently located at fragile sites and genomic regions involved in cancers. Proc Natl Acad. Sci. 101, 2999–3004 (2004). 14973191 10.1073/pnas.0307323101 PMC365734

[b11] Kovalchuk O. *et al.* Involvement of microRNA-451 in resistance of the MCF-7 breast cancer cells to chemotherapeutic drug doxorubicin. Mol Cancer Ther. 7, 2152–2159 (2008). 18645025 10.1158/1535-7163.MCT-08-0021

[b12] Takeshita F. *et al.* Systemic delivery of synthetic microRNA-16 inhibits the growth of metastatic prostate tumors via downregulation of multiple cell-cycle genes. Mol Ther. 18, 181–187 (2009). 19738602 10.1038/mt.2009.207 PMC2839211

[b13] Ma L., Teruya-Feldstein J. & Weinberg R. A. Tumour invasion and metastasis initiated by microRNA-10b in breast cancer. Nature. 449, 682–688 (2007). 17898713 10.1038/nature06174

[b14] Kong A. N., Yu R., Chen C., Mandlekar S. & Primiano T. Signal transduction events elicited by natural products: role of MAPK and caspase pathways in homeostatic response and induction of apoptosis. Arch Pharm Res. 23, 1–16 (2000). 10728649 10.1007/BF02976458

[b15] Hagiwara K. *et al.* Stilbene derivatives promote Ago2-dependent tumour-suppressive microRNA activity. Sci Rep. 2, 314 (2012). 22423322 10.1038/srep00314 PMC3304512

[b16] Burk U. *et al.* A reciprocal repression between ZEB1 and members of the miR‐200 family promotes EMT and invasion in cancer cells. EMBO Rep. 9, 582–589 (2008). 18483486 10.1038/embor.2008.74 PMC2396950

[b17] Spaderna S. *et al.* A transient, EMT-linked loss of basement membranes indicates metastasis and poor survival in colorectal cancer. Gastroenterology. 131, 830–840 (2006). 16952552 10.1053/j.gastro.2006.06.016

[b18] Hurteau G. J., Carlson J. A., Spivack S. D. & Brock G. J. Overexpression of the microRNA hsa-miR-200c leads to reduced expression of transcription factor 8 and increased expression of E-cadherin. Cancer Res. 67, 7972–7976 (2007). 17804704 10.1158/0008-5472.CAN-07-1058

[b19] Park S.-M., Gaur A. B., Lengyel E. & Peter M. E. The miR-200 family determines the epithelial phenotype of cancer cells by targeting the E-cadherin repressors ZEB1 and ZEB2. Genes Dev. 22, 894–907 (2008). 18381893 10.1101/gad.1640608 PMC2279201

[b20] Kota J. *et al.* Therapeutic microRNA delivery suppresses tumorigenesis in a murine liver cancer model. Cell. 137, 1005–1017 (2009). 19524505 10.1016/j.cell.2009.04.021 PMC2722880

[b21] Li Y. *et al.* Up-regulation of miR-200 and let-7 by natural agents leads to the reversal of epithelial-to-mesenchymal transition in gemcitabine-resistant pancreatic cancer cells. Cancer Res. 69, 6704–6712 (2009). 19654291 10.1158/0008-5472.CAN-09-1298 PMC2727571

[b22] Sun M. *et al.* Curcumin (diferuloylmethane) alters the expression profiles of microRNAs in human pancreatic cancer cells. Mol Cancer Ther. 7, 464–473 (2008). 18347134 10.1158/1535-7163.MCT-07-2272

[b23] Singh B. N., Shankar S. & Srivastava R. K. Green tea catechin, epigallocatechin-3-gallate (EGCG): mechanisms, perspectives and clinical applications. Biochem pharmacol. 82, 1807–1821 (2011). 21827739 10.1016/j.bcp.2011.07.093 PMC4082721

[b24] Sharma G., Kar S., Palit S. & Das P. K. 18β‐glycyrrhetinic acid induces apoptosis through modulation of Akt/FOXO3a/Bim pathway in human breast cancer MCF‐7 cells. J. Cell Physiol. 227, 1923–1931 (2012). 21732363 10.1002/jcp.22920

[b25] Jayasooriya R. G. P. T. *et al.* 18β-Glycyrrhetinic acid suppresses TNF-α induced matrix metalloproteinase-9 and vascular endothelial growth factor by suppressing the Akt-dependent NF-κB pathway. Toxicol In Vitro. 28, 751–758 (2014). 24613819 10.1016/j.tiv.2014.02.015

[b26] Fu Y. *et al.* Magnolol inhibits lipopolysaccharide-induced inflammatory response by interfering with TLR4 mediated NF-κB and MAPKs signaling pathways. J. Ethnopharmacol. 145, 193–199 (2013). 23127653 10.1016/j.jep.2012.10.051

[b27] Liu Y. *et al.* The natural compound magnolol inhibits invasion and exhibits potential in human breast cancer therapy. Sci Rep. 3, 3098 (2013). 24226295 10.1038/srep03098 PMC3827615

[b28] Lee H.-S. Rat lens aldose reductase inhibitory activities of Coptis japonica root-derived isoquinoline alkaloids. J. Agric Food Chem. 50, 7013–7016 (2002). 12428952 10.1021/jf020674o

[b29] Vennerstrom J. L. & Klayman D. L. Protoberberine alkaloids as antimalarials. J. Med Chem. 31, 1084–1087 (1988). 3286870 10.1021/jm00401a006

[b30] Hambright H. G., Batth I. S., Xie J., Ghosh R. & Kumar A. P. Palmatine inhibits growth and invasion in prostate cancer cell: Potential role for rpS6/NFκB/FLIP. Mol Carcinog. 10.1002/mc.22192 (2014). PMC4490121 25043857

[b31] You H., Ding W., Dang H., Jiang Y. & Rountree C. B. c‐Met represents a potential therapeutic target for personalized treatment in hepatocellular carcinoma. Hepatology. 54, 879–889 (2011). 21618573 10.1002/hep.24450 PMC3181384

[b32] Ma P. *et al.* Downstream signalling and specific inhibition of c-MET/HGF pathway in small cell lung cancer: implications for tumour invasion. Br J. Cancer. 97, 368–377 (2007). 17667909 10.1038/sj.bjc.6603884 PMC2360323

[b33] Akl M. R. *et al.* Olive Phenolics as c-Met Inhibitors:(–)-Oleocanthal Attenuates Cell Proliferation, Invasiveness, and Tumor Growth in Breast Cancer Models. PLoS One. 9, e97622 (2014). 24849787 10.1371/journal.pone.0097622 PMC4029740

[b34] Kim S. *et al.* MicroRNA miR-199a* regulates the MET proto-oncogene and the downstream extracellular signal-regulated kinase 2 (ERK2). J. Biol Chem. 283, 18158–18166 (2008). 18456660 10.1074/jbc.M800186200

[b35] Li L. *et al.* MiR-34a inhibits proliferation and migration of breast cancer through down-regulation of Bcl-2 and SIRT1. Clin Exp Med. 13, 109–117 (2013). 22623155 10.1007/s10238-012-0186-5

