# Supplementary material for: A robust screening method for dietary agents that activate tumour-suppressor microRNAs
Source: Sci Rep. 2015 Oct 1;5:14697. doi: 10.1038/srep14697 (PMC4589759; doi:10.1038/srep14697)
Supplement: Supplementary Information [file srep14697-s1.pdf]

Supplementary Information

A robust screening method for dietary agents that activate tumour-suppressor microRNAs

Keitaro Hagiwara, [1,2] Luc Gailhouste, [1] Ken Yasukawa, [1,3] Nobuyoshi Kosaka, [1] Takahiro Ochiya[1*]

[1] Division of Molecular and Cellular Medicine, National Cancer Center Research Institute, 5-1-1, Tsukiji, Chuo-ku, Tokyo 104-0045, Japan,

[2] Department of Biological Sciences, Tokyo Institute of Technology, 4259 Nagatsuta-cho, Midori-ku, Yokohama 226-8501, Japan.

[3] Integrative Bioscience and Biomedical Engineering, Graduate School of Science and Engineering, Waseda University, 2-2 Wakamatsu, Shinjuku, Tokyo 162-8480, Japan

[*]Corresponding author

Contents:

1. **Supplementary Figure S1**
2. **Supplementary Table S1**

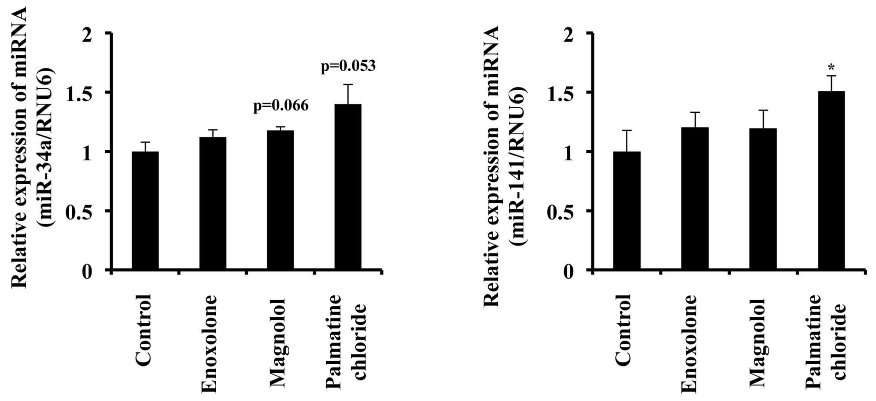

Supplementary Figure S1 - Expression of miR-34a and miR-141 tumour-suppressor miRNAs following natural product treatment.

MCF7 cells were grown and treated with enoxolone, magnolol or palmatine chloride (10 μM), or DMSO (Control). After 2 days of culture, cell extracts were subjected to qRT-PCR. The values on the *y-axis* are depicted relative to the miRNA expression of miR-34a and miR-141. Cells treated with DMSO (Control) were used as controls, and their values were defined as 1.0. All data are shown as the mean ±S.E. *$P<0.05$.

Supplementary Table S1 - miR-200c reporter activity in response to natural product treatment in MCF7 cells. A library containing 139 well-clarified natural substances was utilised.

Natural products	Firefly luciferase activity
Capsaicin	0.84±0.07
(-)-Epigallocatechin gallate	1.00±0.07
(-)-Huperzine A	0.86±0.04
(+)-Usniacin	0.32±0.05
3-Indolebutyricacid	0.89±0.08
4-Methylumbelliferone	0.83±0.10
Aesculin	0.80±0.05
Aloe-emodin	1.39±0.18
Amygdalin	0.28±0.03
Andrographolide	1.02±0.12
Apigenin	5.52±1.37
Arbutin	0.81±0.05
Asiatic acid	0.90±0.01
Aucubin	0.96±0.07
Baicalein	2.44±0.34
Baicalin	0.94±0.15
Bergenin	1.04±0.17
Berberine Hydrochloride	0.35±0.06
β-Sitosterol	1.20±0.09
Bilobalide	1.26±0.19
Caffeic acid	0.90±0.07
Chlorogenic acid	1.04±0.08
Chrysin	7.44±0.44
Cinchonidine	0.98±0.03
Colchicine	0.09±0.01

Cytisine	1.04±0.19
Daidzin	6.40±1.74
Dihydroartemisinin	0.53±0.10
Diosgenin	1.32±0.31
Diosmin	0.78±0.03
DL-Carnitine hydrochloride	0.99±0.02
Ecdysone	1.32±0.01
Emodin	2.64±0.11
Enoxolone	0.75±0.04
Ergosterol	0.80±0.04
Fisetin	1.53±0.27
Formononetin	5.38±1.51
Fumalic acid	1.02±0.10
Genistin	4.21±0.61
Glycyrrhizic acid	1.06±0.05
Gramine	1.05±0.04
Gynostemma Extract	1.25±0.04
Hesperetin	2.02±0.21
Hesperidin	0.90±0.03
Honokiol	1.04±0.09
Hyodeoxycholic acid	1.08±0.15
Icariin	1.02±0.22
Indole-3-carbinol	0.99±0.14
Kaempferol	2.72±0.41
Kinetin	0.82±0.06
L(+)-Rhamnose Monohydrate	1.14±0.08
Limonin	1.09±0.11
Luteolin	3.04±0.20
Magnolol	0.44±0.01
Matrine	0.83±0.09

Methyl-Hesperidin	0.95±0.08
Morin hydrate	1.69±0.22
Myricetin	1.35±0.28
Myricitrin	1.32±0.22
Nalidixic acid	0.97±0.08
Naringin	1.20±0.17
Neohesperidin dihydrochalcone	1.11±0.06
Nobiletin	0.87±0.08
Oleanolic acid	1.06±0.20
Oridonin	1.27±0.30
Orotic acid	1.34±0.22
Osthole	1.17±0.12
Oxymatrine	1.58±0.25
Paeonol	1.07±0.14
Parthenolide	1.03±0.01
Phloretin	1.29±0.08
Phlorizin	1.20±0.06
Piperine	1.92±0.09
Puerarin	0.86±0.07
Quercetin dihydrate	1.40±0.14
Rutaecarpine	3.13±0.67
Rutin	1.08±0.13
Salicin	1.27±0.27
Sclareol	0.86±0.11
Sclareolide	0.72±0.08
Shikimic acid	0.87±0.13
Silibinin	0.94±0.13
Silymarin	0.90±0.17
Sinomenine	0.96±0.10
Stigmasterol	1.06±0.25
Synephrine	1.10±0.21

Tangeretin	1.21±0.33
Tanshinone I	3.08±0.21
Taxifolin	0.92±0.12
Theobromine	0.83±0.11
Tetrahydropapaverine hydrochloride	0.73±0.13
Troxerutin	0.98±0.18
Ursolic acid	0.87±0.14
Vanillylacetone	1.17±0.26
Yohimbine hydrochloride	1.44±0.25
5-hydroxytryptophan	1.29±0.26
Aloin	1.70±0.15
Ammonium Glycyrrhizinate	1.60±0.54
Biochanin A	4.97±0.69
Butylscopolamine bromide	0.97±0.18
Gastrodin	0.97±0.13
ATP	0.89±0.16
Hordenine	0.92±0.06
Indirubin	1.11±0.17
Lappaconite Hydrobromide	1.03±0.24
L-carnitine	1.13±0.24
Naringin Dihydrochalcone	1.25±0.32
Polydatin	9.52±2.19
Quercetin	1.99±0.46
Sesamin	0.51±0.08
Sorbitol	0.91±0.17
Rheochrysidin	1.01±0.14

Salidroside	0.83±0.14
Palmatine chloreide	0.34±0.06
Coenzyme Q10	0.92±0.19
Dihydromyricetin	0.98±0.21
Rhein	1.33±0.21
Sodium Danshensu	1.55±0.31
Cyclovirobuxin D	1.45±0.44
Tetrandrine	0.62±0.14
D-Mannitol	0.71±0.07
Isoliquirtigenin	1.50±0.23
Sophocarpine	0.90±0.15
Chrysophanic acid	1.55±0.36
Curcumol	0.82±0.10
Neohesperidin	0.95±0.14
Xanthone	1.55±0.36
Diosmetin	2.66±0.80
Hematoxylin	1.34±0.37
Naringenin	2.90±0.50
